# Near-death experiences between science and prejudice

**DOI:** 10.3389/fnhum.2012.00209

**Published:** 2012-07-18

**Authors:** Enrico Facco, Christian Agrillo

**Affiliations:** ^1^Department of Neurosciences, University of PadovaPadova, Italy; ^2^Italian Center of Clinical and Experimental HypnosisTorino, Italy; ^3^Department of General Psychology, University of PadovaPadova, Italy

**Keywords:** body-mind problem, consciousness, NDEs, out of body experience, scientific reductionism

## Abstract

Science exists to refute dogmas; nevertheless, dogmas may be introduced when undemonstrated scientific axioms lead us to reject facts incompatible with them. Several studies have proposed psychobiological interpretations of near-death experiences (NDEs), claiming that NDEs are a mere byproduct of brain functions gone awry; however, relevant facts incompatible with the ruling physicalist and reductionist stance have been often neglected. The awkward transcendent look of NDEs has deep epistemological implications, which call for: (a) keeping a rigorously neutral position, neither accepting nor refusing anything a priori; and (b) distinguishing facts from speculations and fallacies. Most available psychobiological interpretations remain so far speculations to be demonstrated, while brain disorders and/or drug administration in critical patients yield a well-known delirium in intensive care and anesthesia, the phenomenology of which is different from NDEs. Facts can be only true or false, never paranormal. In this sense, they cannot be refused a priori even when they appear implausible with respect to our current knowledge: any other stance implies the risk of turning knowledge into dogma and the adopted paradigm into a sort of theology.

During the past decade, an increasing number of studies have focused their attention on the intriguing phenomenon known as “Near-Death Experiences” (NDEs). NDEs are defined as an altered state of consciousness that occurs during an episode of unconsciousness as a result of a life-threatening condition (Moody, 1975). Under these circumstances, patients often report perceiving a tunnel, a bright light, deceased relatives, mental clarity, a review of their lives, and out-of-body experiences (OBEs) in which they describe a feeling of separation from their bodies and the ability to watch themselves from a different perspective (for recent reviews, see Holden et al., 2009; Facco, 2010; van Lommel, 2010; Agrillo, 2011). Most patients describe these experiences as very pleasant, but a few cases may report unpleasant ones (Greyson and Bush, 1992). It is worth noting that the content of NDEs is similar worldwide, across cultures and all times (Belanti et al., 2008). NDEs may occur in people of both genders and all ages, educational and socioeconomic levels, beliefs, and life experiences (Bush, 2002), but a prospective study has reported deeper NDEs in women, in patients having had their first myocardial infarction, those requiring more resuscitation in hospital, and those who have had previous NDEs; the same study reported a lower incidence in the elderly (van Lommel et al., 2001). The exact incidence is not known: taking into account the data from both scientific publications and polls of the general population, the incidence of NDEs can be roughly estimated as between 15% and 20% of critical patients, and some 5% of the general population (Greyson, 1993, 2003; van Lommel et al., 2001). As suggested by some authors (Schroeter-Kunhardt, 1993; Facco, 2010; van Lommel, 2011), the incidence of NDEs has probably increased in the past decades, paralleling the development of techniques of resuscitation and intensive care, which have allowed for a progressive improvement of survival and outcome.

The relatively high incidence and clear phenomenology of NDEs call for scientific explanations of such a complex phenomenon, which appears awkward for its transcendent and sometimes even parapsychological tone. Several neuropsychological and neurobiological hypotheses have been advanced in the past two decades within the ruling context of physicalism and scientific reductionism. Here, we shall shortly outline three items essential for a proper assessment of NDEs: (a) available scientific interpretations; (b) telling facts from hypotheses; (c) epistemological aspects and related scientific prejudices.

## Scientific interpretations

The main hypotheses for NDE interpretations on a scientific basis are: (a) periphery-to-fovea retinal ischemia as a cause of tunnel vision; (b) systemic acidosis and ion shift; (c) temporal lobe dysfunction and epileptic discharges; (d) glutamate-dependent excitotoxic damage and its endogenous modulators (such as agmatine); (e) other neurotransmitter imbalances (including noradrenaline, dopamine, endogenous opioids, serotonin); (f) analogies between NDEs and effects of hallucinogens; (g) REM-sleep intrusions and/or multisensory breakdown involving the right angular gyrus for (OBEs); (h) psychological hypothesis of afterlife expectation.

Centripetal ischemia of the retina has been advocated as the organic cause of tunnel vision, including the observation of syncopal symptoms of pilots flying at G-force acceleration (Whinnery and Whinnery, 1990). A visual cortex dysinhibition associated with anoxia has also been postulated as an explanation for tunnel-like perception (Blackmore and Troscianko, 1988; Blackmore, 1996). High concentrations of carbon dioxide (CO_2_) and/or hyperkalemia also have been advanced as an explanation for some of the recurring features of NDEs (Meduna, 1950; Klemenc-Ketis et al., 2010). Since endogenous opioids are released under stress, as during hemorrhagic shock (Molina, 2003), they have been postulated as a possible mechanism for the positive emotional tone of NDEs. Likewise, the excitotoxic brain damage yielded by uncontrolled glutamate release in acute brain lesions led Jansen to speculate its role in NDE origin and consider ketamine administration as a model for NDEs and OBEs (Jansen, 1989, 1990, 2000).

Britton and Bootzin (2004) investigated temporal lobe function in patients reporting NDEs and reported a higher rate of temporal lobe epileptiform discharges in the NDE group in comparison to controls, suggesting that a temporal lobe dysfunction may underlie NDEs; likewise, in a review of autoscopic hallucinations due to focal brain damage, a tendency of OBEs toward a higher rate of right temporal lobe lesions was found (Blanke and Mohr, 2005). REM-sleep intrusions and sleep paralysis associated with hypnagogic and hypnopompic experiences have also been advocated as causes of NDEs and OBEs (Cheyne et al., 1999; Nelson et al., 2006); in a retrospective study, a higher rate of these experiences was reported in subjects with NDE than in controls (Nelson et al., 2006).

The neurophysiological mechanisms of OBEs have been more widely investigated and also partially reproduced in the laboratory. Their first interpretation was in terms of autoscopy, a well-known psychiatric symptom, but the features of OBEs are entirely different from classical, psychiatric descriptions of autoscopy (Brugger et al., 1997; Brugger and Mohr, 2009), as well as from depersonalization disorders. Interestingly, Ehrsson (2007) induced an illusion of being outside the physical body in healthy participants by manipulating both visual and tactile perceptions, suggesting that this kind of experience may result from the combination of visual perspective and other related multisensory information. Even though extensive debate surrounds the nature of these induced experiences—sometimes considered only “illusionary experiences” along the lines of bodily illusions, rather than real “out of body” experiences (Greyson et al., 2009; van Lommel, 2011)—it seems plausible that such experiences may be mediated by specific brain regions, where the right angular gyrus might probably play a primary role (Blanke et al., 2004; Blanke and Arzy, 2005; De Ridder et al., 2007; Lopez et al., 2008).

Psychological interpretations of NDEs mainly regard the “expectation hypothesis”: according to it, NDEs would be the product of altered mental states yielded by life-threatening conditions (Blackmore and Troscianko, 1988; Appleby, 1989; French, 2001; Britton and Bootzin, 2004), which would trigger NDE phenomenology as a projection of beliefs and expectancy of the afterlife. In this sense, Christians would be likely to see Jesus in the light, while Muslims might see Allah. Also, atheists are supposed to be tangled in the same cognitive mechanism, with people projecting their wishes to be rejoined with their own deceased relatives. Other psychological interpretations encompass the memory of being born, depersonalization, dissociation, personality factors, fantasies, and imagination (French, 2005; Greyson et al., 2009; van Lommel, 2010). Very little evidence, however, supports these latter interpretations.

## Telling facts from fallacies

The above-mentioned studies received a lot of media coverage because they have undoubtedly provided useful information in the process of understanding at least some of the recurring features of NDEs, but it should be recognized that most if not all interpretations remain only speculation or, at best, clues of the possible brain mechanisms triggering them; some of the results seem questionable or even odd, taking into account other well-known clinical facts:

- In a sudden severe acute brain damage event such as cardiac arrest, there is no time for an experience of tunnel vision from retinal dysfunction, given that the brain is notably much more sensitive to anoxia and ischemia than peripheral organs; its role in coma from acute brain lesions (such as trauma or hemorrhage) is also questionable, as the pathophysiology of brain damage does not imply retinal ischemia. Fainting due to arterial hypotension—a common event—does not seem to be associated with the tunnel visions described in NDEs. In a comprehensive review of symptoms and signs of syncope (Wieling et al., 2009), the prodromal visual changes were described as blurred and fading vision, scotomas, color changes, dimming or graying of the peripheral field of vision (“graying out”), followed by peripheral light loss and complete blindness (“blacking out”). Graying out has been clearly described in experimental conditions only, such as during exposure to centrifugal force. There may be a link between graying out and the experience of seeing a tunnel, but the latter is qualitatively different and seems to depend on cultural factors as well (Belanti et al., 2008): in fact, it is usually described as passing through a tunnel and reaching a new landscape (van Lommel et al., 2001; Facco, 2010), while graying out is a much simpler transient sensation usually followed by blackout. These data as a whole make the retinal hypothesis as the main mechanism of tunnel vision plausible at best only for pilots and falls from a high altitude in the mountains.
- Endogenous opioids, which are likely released in critical conditions, are only weak hallucinogens, though they might help to evoke vivid experiences, particularly when in combination with cognitive confusion. Nevertheless, NDEs are not reported by patients using opioids for severe pain, while their cerebral adverse effects display an entirely different phenomenology in comparison to NDEs (Mercadante et al., 2004; Vella-Brincat and Macleod, 2007). Morse also found that NDE occurrence in children is independent from drug administration, including opioids (Morse et al., 1986). Therefore, opioids are far from successful at entirely explaining the positive mood and vivid “hallucinations” of NDEs.
- The topic of neurotransmitter imbalance and hallucinogens is very complex and far beyond the limits of this analysis; however, even though some psychedelic drugs such as DMT and ayahuasca can give rise to quite similar experiences (Strassman, 2001), aside from providing usable analogies for NDEs, there are marked differences between the hallucinations that accompany use of psychedelic drugs and NDEs, preventing the latter's interpretation as a simple byproduct of the release of specific neurotransmitters (see Facco, 2010, as a review of the topic). The closest similarity is seen in shamanic or religious rituals using specific agents, such as the use of iboga in the Bwiti religion in Gabon (Strubelt and Maas, 2008); anyway, it must be taken into account that cultural factors such as ritual, personality, environment, and aims for hallucinogen consumption are no less relevant than the agent itself with regard to the content and meaning of the experience, which is largely variable for any drug.
- Brain lesions, the excitotoxic damage, and the whole of pharmacologic side effects of therapy (including opioids, steroids, and anticholinergic agents) may yield a picture of delirium: this condition is well known in anesthesiology and intensive care, but both its clinical picture and content of experience differentiate it entirely from NDEs (Facco and Rupolo, 2001; Xie and Fang, 2009; Frontera, 2011).
- Of the two mentioned studies on the temporal lobe (Britton and Bootzin, 2004; Blanke and Mohr, 2005), one was retrospective and included cases with focal brain damage only, while in the other, the control group was made up of participants without any history of life-threatening illness or injury. According to the scientific principle of isolating the independent variable, the two compared groups should have been exactly the same except for the investigated variable (i.e., presence/absence of NDEs)—that is, patients with life-threatening events should be comparable to patients reporting NDEs. Therefore, in Britton and Bootzin's study (2004), the tendency toward a temporal lobe dysfunction in patients reporting NDEs, though of interest, might simply be the result of the injury, without any cause-effect relationship with NDEs.
- The hypothesis of REM intrusions (Nelson et al., 2006) is not compatible with cardiac arrest, a condition in which brain electrical activity is silent, though it may remain an interesting neurophysiological aspect of experiences occurring in non-critical conditions. Also in the study by Nelson et al. (2006) the control group was made up of participants without life-threatening events, thus making any rigorous comparison with the experimental group impossible. Above all, Greyson et al. (2009) noted that researchers did not ask whether the REM intrusion symptoms occurred before or after NDEs. In this sense, it might be equally possible that NDEs determine subsequent REM intrusions (instead of the latter being the cause of NDEs).
- The changes in CO_2_ and kalemia have not been confirmed in other studies (Parnia et al., 2001), but these two parameters might be related to NDEs as possible triggers for the events or for the capability to recall them (Greyson, 2010a). Anyway, it should be taken into account (as with any other factors) that even if these two parameters may have a role in triggering NDEs, the content and meaning of NDEs do not specifically depend on any substance.
- Neurobiological interpretations of NDEs imply that brain disorders are a *sine qua non* condition for these experiences, thus excluding their occurrence in physiological conditions. On the contrary, near-death-like experiences have been reported in the absence of cerebral dysfunctions (Owens et al., 1990; Gabbard and Twemlow, 1991; Facco and Agrillo, under revision). To that effect, van Lommel (2010) summarized some of the most frequently recurring circumstances that might prompt NDEs in the absence of brain function disorders. These include serious (but not immediately life threatening) conditions, isolation, depression, existential crisis, meditation, and similar experiences (the so-called “fear-death experiences”). Another potential circumstance was described by Moody and Perry (2010), who reported shared death experiences in healthy people attending the moment of death of a close relative. These kinds of experiences represent a further challenge to the above-mentioned reductionistic and mechanistic interpretations, given that they are unrelated to brain disorders.
- Evidence against simple mechanistic interpretations comes also from a well-known prospective study by van Lommel et al. (2001), which showed no influence of given medication even in patients who were in coma for weeks. Factors such as duration of cardiac arrest (the degree of anoxia), duration of unconsciousness, intubation, induced cardiac arrest, and the administered medication were found to be irrelevant in the occurrence of NDEs. Also, psychological factors did not affect the occurrence of the phenomenon: for instance, fear of death, prior knowledge of NDE, and religion were all found to be irrelevant. Above all, only 12% of patients had a core experience: if physiological and psychological factors were the cause of NDE, most of the patients would be expected to report it.

## Epistemological implications, related scientific prejudices and neglected facts

NDEs are an intriguing and relevant phenomenon, the nature of which is still under debate. Their apparent trascendent tone may wrongly lead one to take them as clues of an afterlife, glossing over the neurobiological mechanisms involved in producing them; on the other hand, a prejudicial refusal of facts that appear trascendent or paranormal might wrongly lead to neglecting them due to their apparent incompatibility with the widely accepted materialistic view of the world and known scientific laws. Both these stances may be harmful sources of opposite errors, the former leading to belief in non-existing “facts,” the latter to denial of existing ones. To illustrate this principle, consider that ancient Chinese astronomers, whose cosmological beliefs did not preclude celestial change, recorded the appearance of new stars much earlier than Western astronomers who believed in an immutable heaven (Kuhn, 1970).

The available scientific explanations are very relevant in the process of understanding NDEs, but they still remain hypotheses, given the persistent lack of proofs. There is a need for further efforts undertaken with an open mind and a truly skeptical stance (that is, neither accepting nor refusing any possibility *a priori*) to avoid the risk of putting belief and faith (not just scientific ones) before facts, with the implicit risk of giving rise to new wrong beliefs and dogmatic drifts. According to van Lommel (2010), “*true science does not restrict itself to narrow materialistic assumptions but is open to new and initially inexplicable findings and welcome the challenge of finding explanatory theories*” (p. 331).

In a recent prejudicially skeptical review, Mobbs and Watt (2011) provided a synthetic outline of possible neurobiological mechanisms of NDEs, concluding that there is nothing paranormal about them. This statement implies a clear-cut incompatibility between science and parapsychology, which is at least partly questionable. In fact, parapsychology may be defined as the study of physical phenomena beyond those presently understandable (Morris, 2001)—a matter that in itself does not imply any incompatibility with science and its methodologies. Instead, it only tracks the border between what is actually known/understandable and what is still to be understood/redefined, while facts by themselves can only be true or false, not paranormal. The same goes for religious visions, mentioned in the Mobbs and Watt (2011) paper in terms of delusions or hallucinations only, in order to justify a dysfunctional origin of NDEs' transcendent components. Brain disorders may yield religious delusions, but taking into account only delusions without considering the possibility of true and meaningful religious experiences (with their deep psychological, philosophical, and cultural implications) may be misleading, like a false syllogism.

In the tricky inductive process that aims at understanding new phenomena, two main steps can be recognized: (a) explaining them on the basis of what is known; (b) discovering new laws that allow for explanations. The former is the simpler approach, and the first recourse; the second is much more difficult and is entered when available explanations are not able to fit facts. During this tricky phase, unavoidably spurious arguments are often introduced in attempts to explain new facts with old theories; instead, one should welcome evidence that is able to falsify given theories, in order to test the viability of such evidence (Popper, 1963). Such a problem occurred in the process of arriving at a definition of brain death over a period of some 20 years, from the 1960s to 1980s (see Facco, 2001, for further details). This might also be the case for NDEs, the available scientific interpretations of which are far from fitting NDE phenomenology.

As already discussed, the idea that NDEs are the mere results of a brain function gone awry looks to rely more on speculation than facts (Mobbs and Watt, 2011) and suffers from bias in skipping both the facts and hypotheses that challenge the reductionist approach (e.g., see van Lommel, 2004, 2011; Facco, 2010; Greyson, 2010b; Agrillo, 2011). Simple advocated physical causes, such as anoxia/ischemia, explain very well the common experience of fainting, but are far from explaining the nature of NDEs or why NDEs occur in only a minority of cases, as already emphasized by van Lommel et al. (2001). Furthermore, complete brain anoxia with absent electrical activity in cardiac arrest is incompatible with any form of consciousness, according to present scientific knowledge, making the finding of an explanation for NDEs a challenging task for the ruling physicalist and reductionist view of biomedicine (Kelly et al., 2007; Greyson, 2010b; van Lommel, 2010). In order to safeguard the accepted axioms, odd comments have sometimes also been reported. For instance, in order to justify the occurrence of NDEs, Bardy (2002) questioned whether in cardiac arrest with flat EEG brain electrical activity is really silent; however, it is well known that this is not the case (Parnia and Fenwick, 2002).

There is increasing evidence that consciousness is mediated by a large-scale coherence in the gamma band, binding different cortical areas, and recurrent activity between the cortex and thalamocortical loops, with perceptual periods in the order of 80–100 msec (Singer, 1998, 2001; Zeman, 2001; John, 2002; Melloni et al., 2007). Anesthesia can suppress consciousness by simply interrupting binding and integration between local brain areas without the need for suppressing EEG activity (Alkire and Miller, 2005; Alkire et al., 2008). This is the reason why, in clinical practice, general anesthesia can be associated with almost normal EEG with peak activity in the alpha band (Facco et al., 1992), while in deep, irreversible coma, consciousness can be lost even with a preserved alpha pattern activity (Facco, 1999; Kaplan et al., 1999). In short, loss of consciousness can occur with preserved EEG activity, while, in the case of a flat EEG, neither cortical activity nor binding can occur; furthermore, short latency somatosensory-evoked potentials, which explore the conduction through brain stem up to the sensory cortex and are more resistant to ischemia than EEG, have been reported to disappear during cardiac arrest (Yang et al., 1997). The whole of these data clearly disproves any speculation about residual undetected brain activity as a cause for some conscious experience during cardiac arrest.

A few well-witnessed cases of NDEs suggest the possibility of a partial dissociation between body and mind (Sabom, 1998; van Lommel et al., 2001; van Lommel, 2011): they sound odd and hardly compatible with our present knowledge, but might be a clue of possible, still unknown properties of consciousness. Even the oddest facts, if true, should not be neglected but rather received with an open mind and investigated for the sake of coherence with the essence of scientific knowledge.

Finally, the data available in the literature are not easily compatible with the interpretation of “meeting deceased people” as a mere consequence of the psychological hypothesis of expectation, considering that in most cases the perception of undefined entities (not belonging to the iconography of the patients' religion) and figures other than known deceased persons has been reported (see, for instance, Holden et al., 2009; van Lommel, 2010). Moreover, it is unclear how people in sudden critical conditions (such as cardiac arrest) might be aware of being near-death and have time enough to develop complex scenarios according to their wishes. Also the occurrence of NDEs in children, even as young as three year old (Morse et al., 1985, 1986), does not support an expectation hypothesis, given their lack of a clear vision of death and of elaborate philosophical-religious views of life.

The neurobiological correlations between NDEs, the parieto-temporo-occipital junction (see Lopez et al., 2008, as a review of the topic), the limbic system (Blackmore, 1996), and the temporal lobe (Britton and Bootzin, 2004) are relevant; however, it is widely known that statistical correlations of mental and biological processes do not imply that the former totally derive from the latter and do not prove any cause-effect relationship between the two. Exactly as our legs are the substrate or correlate of walking, neural networks are necessary for mental phenomena, but this does not imply we decide to run because of legs (Bunge, 2010). Even assuming a casual relation, which is not the case, abnormal activity in the temporal lobe or other locations might be sufficient for the occurrence of some features of NDEs, but concluding that such pattern activities are necessary for NDEs is another thing, not yet demonstrated. In this regard, Britton and Bootzin (2004) in the above-mentioned study on NDEs and the temporal lobe correctly admitted that the differences observed between NDEs and the control group were probably the generalized result of trauma rather than specific to NDEs. Last but not least, Mobbs and Watt (2011) provided an appealing analogy between NDEs and some mental disorders, such as the Cotard syndrome. Nevertheless, from a phenomenological point of view, the Cotard syndrome looks to be just the opposite of NDEs: the former is a delusion of being dead when alive, while the latter is the awareness of being conscious and alive when clinically dead (i.e., in cardiac arrest with flat EEG). Such an analogy is not acceptable and, anyway, analogy does not imply identity; even assuming an identity of the kind of experience, both its meaning and pathophysiology might be totally different.

In conclusion, NDEs are an intriguing and still misunderstood phenomenon, challenging the heart of neurobiological axioms (i.e., the idea of consciousness as an epiphenomenon of brain circuitry). In this regard we should keep in mind that the study of consciousness has been *a priori* rejected by Galilean sciences and relegated to philosophy and religion for centuries: this was not the result of a free and well-founded epistemological reflection but a byproduct of the conflict with the Inquisition, being that the soul (that is, psyche and mind) was an exclusive matter of theology. As a result, the study of consciousness has become one of the main topics of neuroscience only in recent years and is still in its very beginning stages; we probably know much less about the mind than we are inclined to believe, despite the wealth of valuable data on neuroimaging of brain functions. Even worse is our knowledge of spirituality and other still misunderstood mind activities (the so-called altered states of consciousness), including NDEs, hypnosis, meditation, and mystic experiences (Vaitl et al., 2005). As far as spirituality is concerned, its very name is a source of mistrust in the world of materialistic science (due to the above-mentioned historical reasons): here, it is only worth emphasizing that spirituality is a faculty of the mind, and, as such, it is independent from any theological or doctrinal view and can be scientifically studied [see the outstanding recent books by Kelly et al. (2007) and Walach et al. (2011)]. It is now time to remove the ongoing cultural filters and include consciousness, spirituality, and the highest mind expressions in neuroscience in a free, secular, and scientific perspective to overcome old prejudices.

The value of neurobiological and reductionist approaches is not discussed here, but only their possible wrong use (i.e., when their assumptions are taken for absolute, unchanging truths, likewise the dogmas of theology and catechism). In fact, the essential virtue of modern science is twofold: (a) the capability to gain systematic knowledge of the physical world through observation, experimentation and a critical process of falsification; (b) the capability of reforming its own theories and even axioms and language, when the accepted model of reality turns out to be incompatible with facts, leading to the “paradigm shifts” claimed by Kuhn (1970). Such a dramatic shift occurred in the twentieth century, when relativistic and quantum physics overturned classical thought.

Reductionism is a good and powerful tool we should make a good use of, but it is only a tool, like a knife, which can be used for both saving a life or killing a man. The reductionist approach is essential for studying areas of the brain and mechanisms involved in specific functions, but it looks to be blind to the phenomenality of experiences, meanings, values, and their impact on human life and culture, which remain on the dark side of the reductionistic moon. Here it is only worth mentioning how the relationship between mind and brain, the so-called “hard problem,” is still an unsolved problem (Chalmers, 1995, 1999; Rudrauf et al., 2003; Ibanez, 2007). The whole of data here reported indicates an increasing need for a broader scientific approach to consciousness and other non-ordinary activities of mind, including those belonging to the suspicious areas of transcendence and spirituality, with their still misunderstood physiology. This might be the case with NDEs as well, where taking *a priori* the content of such awkward experiences as exclusive expression of brain pathology and worthless epiphenomena of brain circuitry might lead to misleading results.

### Conflict of interest statement

The authors declare that the research was conducted in the absence of any commercial or financial relationships that could be construed as a potential conflict of interest.
